# Supplementary material for: A latitudinal gradient in Darwin’s naturalization conundrum at the global scale for flowering plants
Source: Nat Commun. 2023 Oct 12;14:6244. doi: 10.1038/s41467-023-41607-w (PMC10570376; doi:10.1038/s41467-023-41607-w)
Supplement: Supplementary file 1 — Supplementary Information [file 41467_2023_41607_MOESM1_ESM.pdf]

Supplementary Information for

A latitudinal gradient in Darwin's naturalization conundrum at the global scale for vascular plants

Shu-ya Fan[†], Qiang Yang[†], Shao-peng Li[*], Trevor S. Fristoe, Marc W. Cadotte, Franz Essl, Holger Kreft, Jan Pergl, Petr Pyšek, Patrick Weigelt, John Kartesz, Misako Nishino, Jan J. Wieringa & Mark van Kleunen

[†] These authors contributed equally to this work.

[*] Correspondence to: spli@des.ecnu.edu.cn

This file includes:

Supplementary Table 1 to 4

Supplementary Figure 1 to 14

Supplementary Table 1. Normalization functions used and loadings of the 19 bioclimatic variables on the first two axes of the principal component analysis (PCA).

Bioclimatic variables	Transformation function	D value of Kolmogorov-Smirnov test	Loadings	
			PC_{Temp}	PC_{Prec}
Annual mean temperature	inverse log	0.09	0.32	-0.13
Annual precipitation	cube root	0.03	0.19	0.32
Isothermality	boxcox	0.08	0.31	-0.03
Maximum temperature of warmest month	boxcox	0.06	0.23	-0.23
Mean diurnal range	raw	0.03	0.05	-0.29
Mean temperature of coldest quarter	inverse log	0.09	0.33	-0.04
Mean temperature of driest quarter	inverse log	0.09	0.28	-0.12
Mean temperature of warmest quarter	inverse log	0.05	0.23	-0.23
Mean temperature of wettest quarter	inverse log	0.07	0.24	-0.15
Minimum temperature of coldest month	inverse log	0.07	0.33	-0.002
Precipitation of coldest quarter	boxcox	0.04	0.09	0.32
Precipitation of driest month	cube root	0.11	0.02	0.35
Precipitation of driest quarter	boxcox	0.06	0.02	0.37
Precipitation of warmest quarter	sqrt	0.02	0.10	0.31
Precipitation of wettest month	boxcox	0.03	0.21	0.25
Precipitation of wettest quarter	boxcox	0.03	0.20	0.27
Precipitation seasonality	sqrt	0.01	0.09	-0.22
Temperature annual range	cube root	0.04	-0.30	-0.09
Temperature seasonality	sqrt	0.05	-0.32	-0.03
Eigenvalue			8.68	6.03
Proportion of variance			45.69%	31.73%
Cumulative proportion			45.69%	77.42%

Columns from left to right: name of the bioclimatic variable; transformation function that made the variable best approximate a normal distribution; D value of the Kolmogorov-Smirnov test for the best transformation; and loadings on the first two PCA axes.

Supplementary Table 2. The references of native species list for the regions not covered by the Global Inventory of Floras and Traits (GIFT) database at the time of data extraction. The IDs for each region correspond to the IDs used in the open-access GloNAF database (see https://idata.idiv.de/DDM/Data/ShowData/257).

ID of region	Name of region	References
353	Papua New Guinea	Cámara-Leret, R. et al. New Guinea has the world's richest island flora. *Nature* **584**, 579–583 (2020).
919; 952	Israel; Moldova	Euro+Med (2006-): Euro+Med PlantBase - the information resource for Euro-Mediterranean plant diversity. Published on the Internet http://ww2.bgbm.org/EuroPlusMed/ [accessed *2019/12/30*].
1818	India	Govaerts R (ed.). 2022. WCVP: World Checklist of Vascular Plants. Facilitated by the Royal Botanic Gardens, Kew. https://doi.org/10.34885/nswv-8994 [accessed 27 October 2022].

Supplementary Table 3. The influence of latitude on mean pairwise phylogenetic distance (MPD) between naturalized and native plant species, analyzed with different minimum region-size thresholds. The relationships were estimated with linear models.

	Region size thresholds	Number of regions	Estimate	Std. Error	t value	Pr(>|t|)	Adjusted R-squared
1	0	611	-0.095	0.011	-8.679	<0.0001	0.109
2	1,000km^2	508	-0.105	0.009	-11.596	<0.0001	0.208
3	5,000km^2	487	-0.101	0.009	-11.272	<0.0001	0.206
4	10,000km^2	466	-0.100	0.009	-11.117	<0.0001	0.209

Supplementary Table 4. The influence of latitude on mean pairwise phylogenetic distance (MPD) between naturalized and native plant species, analyzed with different subsets of regions. The relationships were estimated with linear models (1) for all regions, (2) for the subset of regions where the completeness of naturalized species lists exceeded 50%, and (3) for the subset of regions for which average inventory-completeness percentages were available and weighted by the inventory-completeness percentage.

Models	Regions' number	Estimate	Std. Error	t value	Pr(>|t|)	Adjusted R-squared
(1)	487	-0.101	0.009	-11.272	<0.0001	0.206
(2)	467	-0.098	0.009	-10.780	<0.0001	0.198
(3)	430	-0.101	0.010	-9.563	<0.0001	0.174

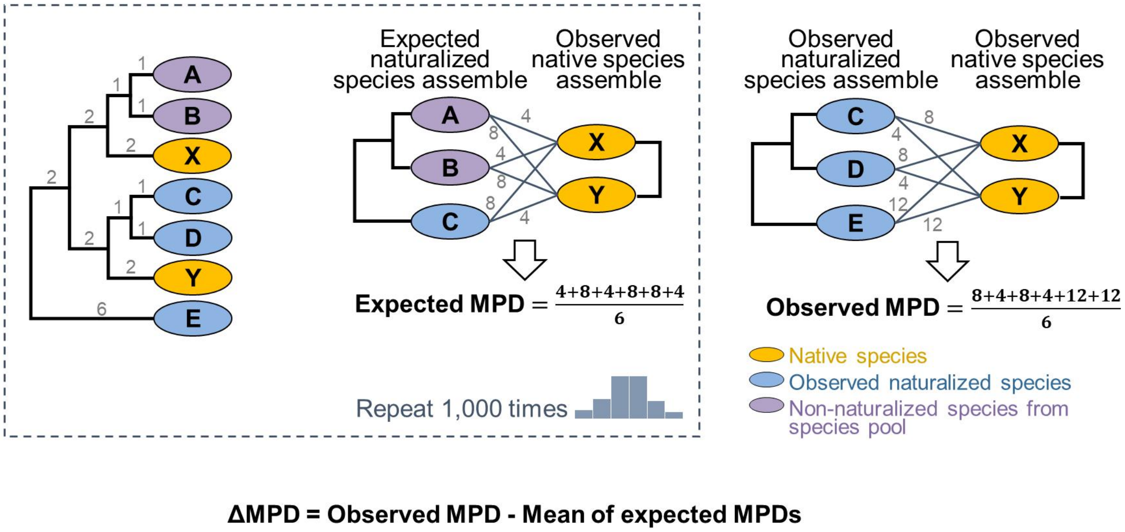

Supplementary Fig. 1. Diagram illustrating the calculation of the observed MPD between naturalized alien and native plant species, as well as the difference (ΔMPD) between the observed MPD and the mean of the expected MPDs generated by the null model.

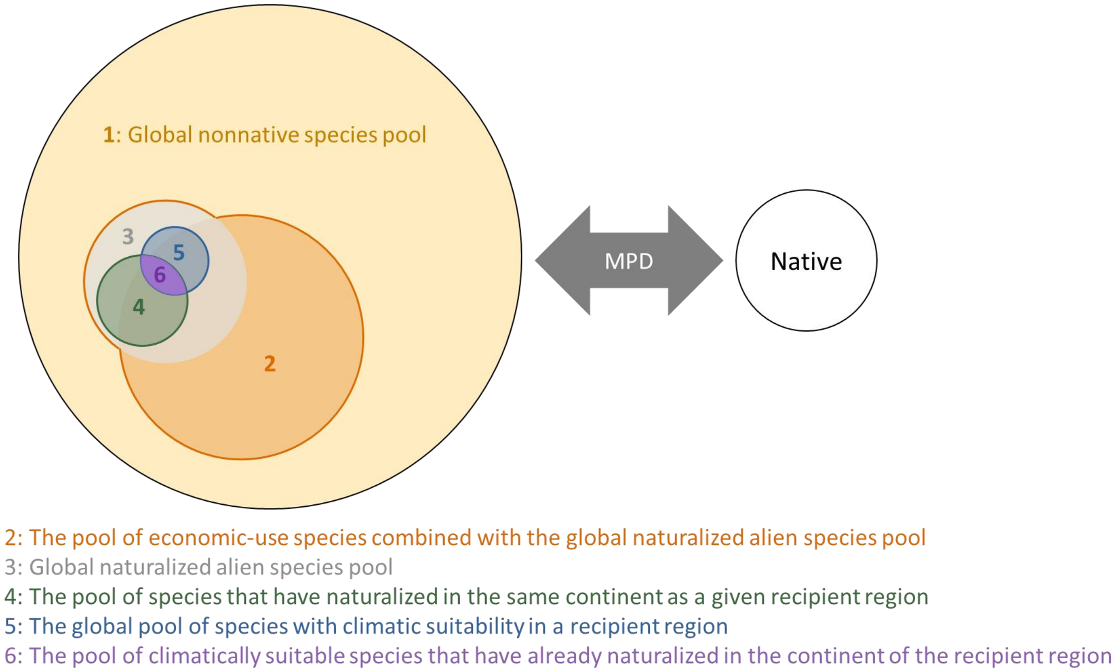

Supplementary Fig. 2. Schematic representation of the six different potential naturalized species pools used in the null models for calculating the expected MPDs of alien species to the native species in a region. The black-rimmed circles jointly indicate the global flora, with the species that are native to a focal region in the white circle and the species that are not native there in yellow. The latter form the global nonnative species pool (1; global nonnative flora). Some of those alien species, indicated by the orange outline, are known to have economic uses (and therefore likely to be cultivated outside their native ranges; orange circle) or to be naturalized somewhere in the world (gray circle). Together they form the pool of economic-use species combined with the global naturalized alien species pool (2; econ. use flora). The next species pool is the global naturalized alien species pool, which include species that have naturalized somewhere in the world (3; global nat.; gray circle). Another species pool is the subset of naturalized species that have naturalized in the same continent as a given recipient region (4; continent nat.), indicated by the green circle with green outline. Another subset of naturalized species occurs in regions with similar climates as the focal region and form the

global pool of species that are climatically suitability for the recipient region (5; climate nat.), indicated by the blue circle with blue outline. The final subset, indicated by the purple overlap of the green and blue circles, is the intersection of the two preceding species pools (4 and 5), which consist of species that are climatically suitable for the region and have already naturalized in the continent of the recipient region (6; climate continent nat.).

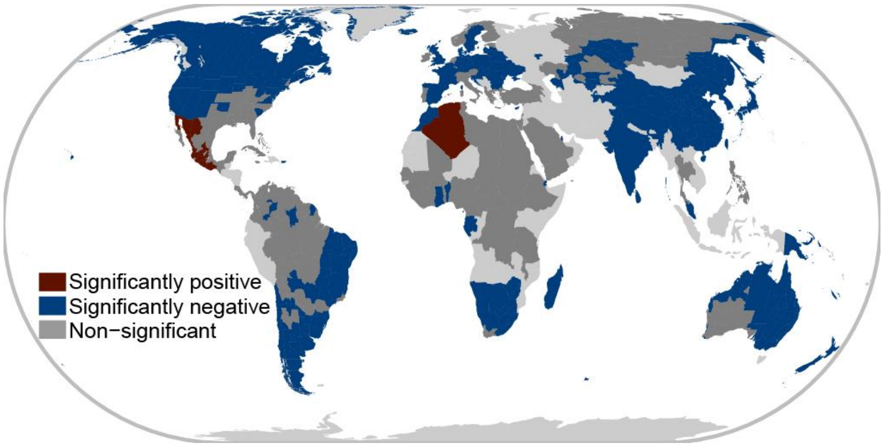

Supplementary Fig. 3. Statistical significance of the observed MPD compared to the expected MPD in 487 regions around the world. The blue, red, and dark gray areas represent regions where the observed MPD values are statistically significantly lower, higher, or non-significantly different, respectively, from the expected MPDs at thresholds of $P < 0.05$. The expected MPD values were generated based on the null model that used the global naturalized alien species pool. Gray areas indicate regions without available data.

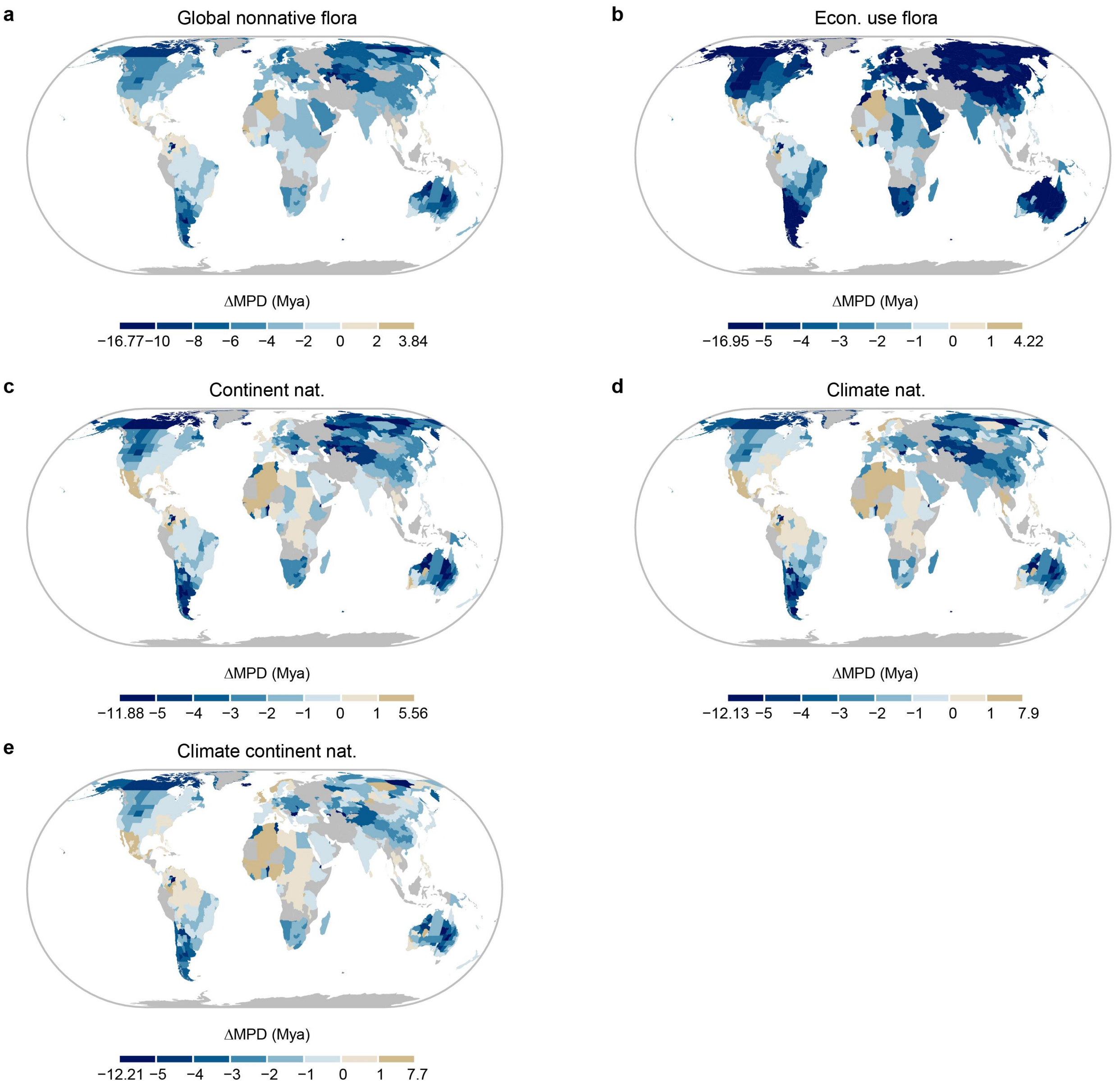

Supplementary Fig. 4. Global maps displaying the distribution of ΔMPDs derived from 1,000 randomizations using five different types of null models across 487 regions. ΔMPDs were calculated using five different species pools (**a,** global nonnative flora; **b,** econ. use flora; **c,** continent nat.; **d,** climate nat.; **e,** climate continent nat.) as shown in Supplementary Fig. 2. Gray areas are regions without data.

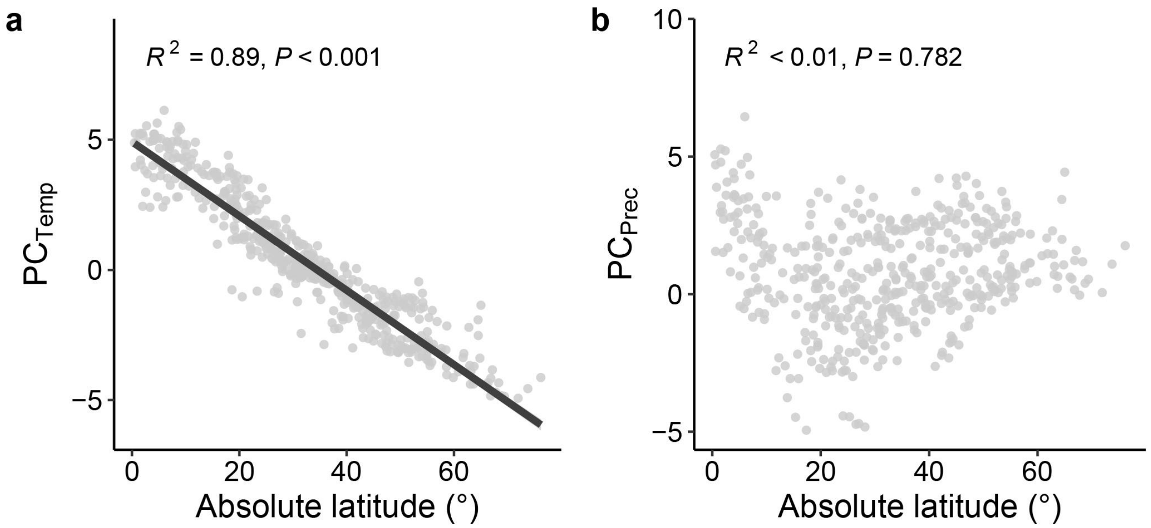

Supplementary Fig. 5. Latitudinal patterns of the first two climactic principal components (a, PC_{Temp}; b, PC_{Prec}) for the 487 regions. Lines represent fitted relationships from linear regression models, and shaded areas represent the 95% confidence intervals. The displayed R^2 values have been adjusted.

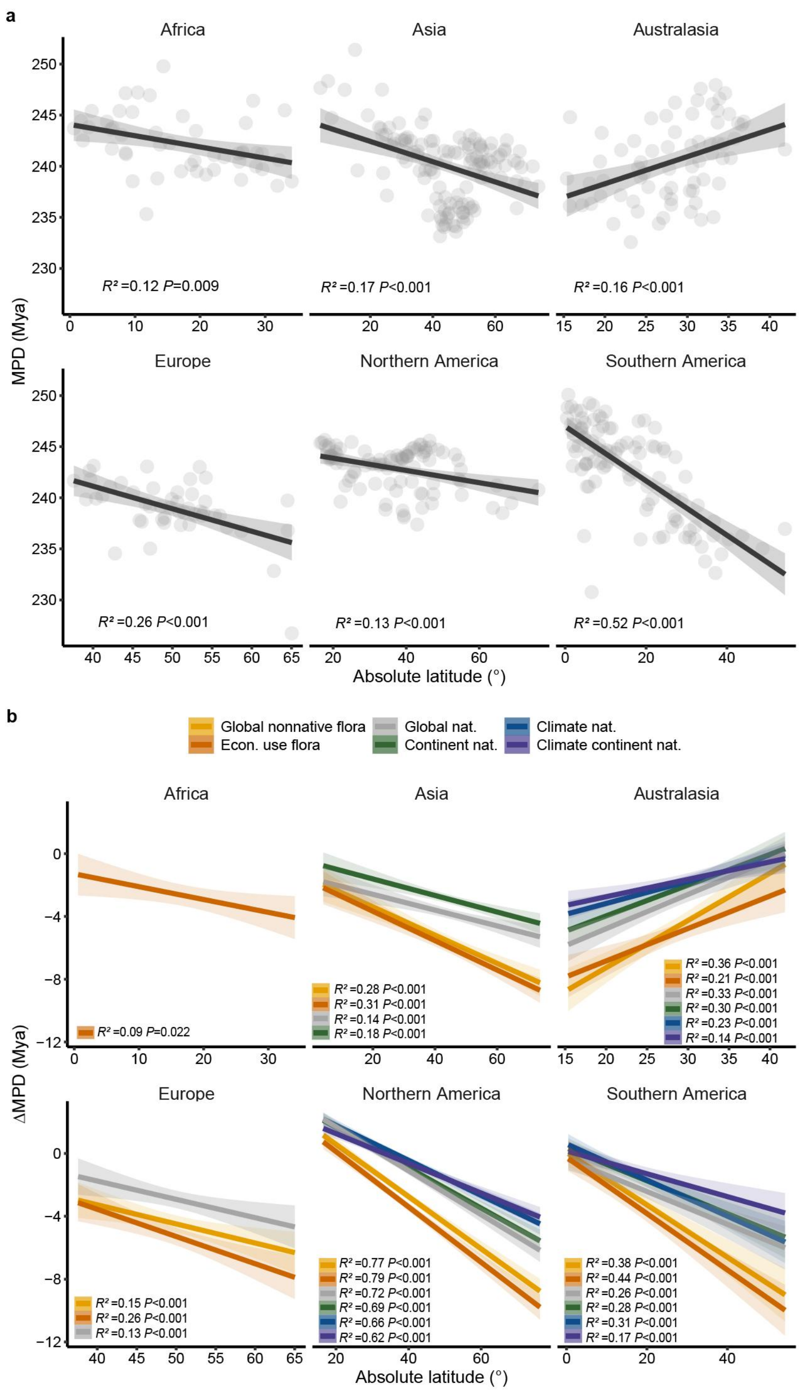

Supplementary Fig. 6. Latitudinal gradients of observed MPD (a) and ΔMPD (b) by continent in 484 regions. In **b**, different colors represent different species pools used to calculate ΔMPD (Supplementary Fig. 2). The lines and shaded areas represent predictions and their 95% confidence intervals obtained from linear models of ΔMPD on absolute latitude. All R^2 values have been adjusted, and non-significant ($P >= 0.05$) relationships are not shown.

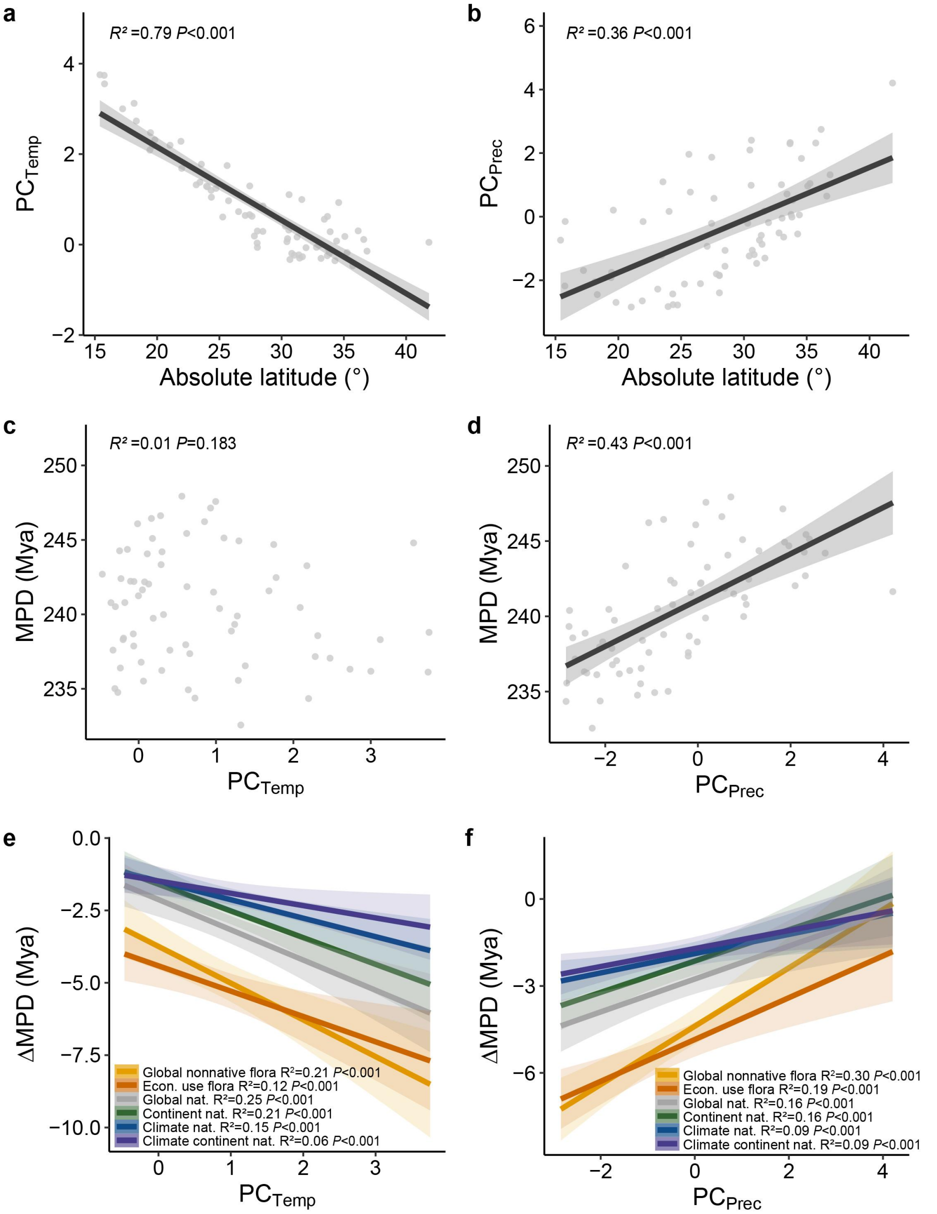

Supplementary Fig. 7. Relationships between climate variables and latitude (a, PC_{Temp}; b, PC_{Prec}), as well as phylogenetic distance metrics and climate variables, of 69 regions in Australasia. **c** and **d** show the relationships of MPD with PC_{Temp} and PC_{Prec}, respectively. **e** and **f** show the relationships of ΔMPD with PC_{Temp} and PC_{Prec}, respectively. In **e** and **f**, different colors represent different species pools used to calculate ΔMPD (Supplementary Fig. 2). Lines and shaded areas represent predictions and their 95% confidence intervals, respectively. All R^2 values have been adjusted, and non-significant (P >= 0.05) relationships are not shown.

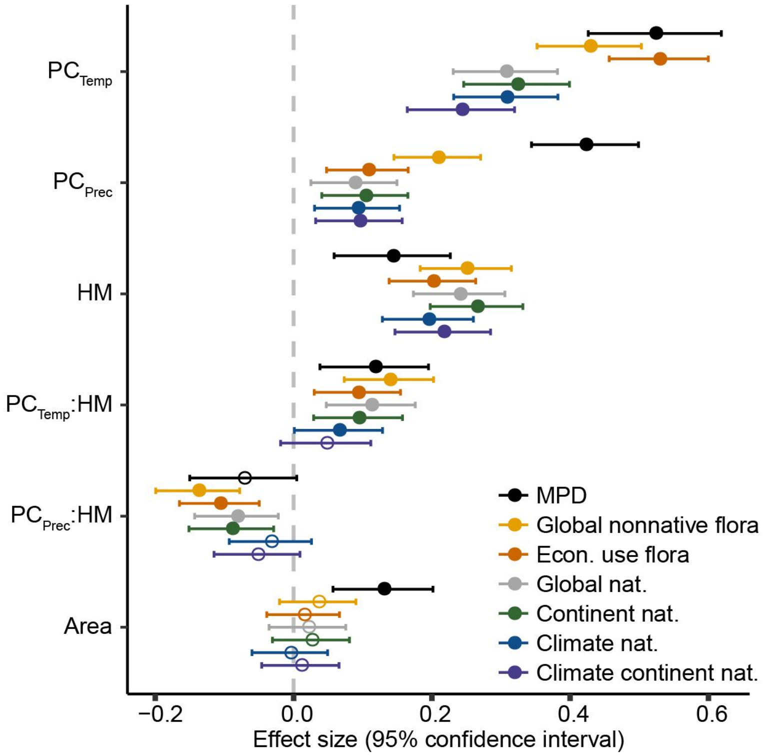

Supplementary Fig. 8. The effects of climate, human environmental modification and region area on phylogenetic distance metrics between naturalized alien and native species in the 487 regions. The effect sizes of climate variables, human modification (HM), their interactions, and region area on observed MPD and ΔMPD are represented by points and error bars, estimated using linear mixed-effects models. Different colors represent different species pools used to calculate ΔMPD (Supplementary Fig. 2). The circles represent the coefficient estimates and the error bars represent the 95% confidence interval. Solid circles denote statistically significant effects, while open circles represent non-significant effects. Note that the area of the region was log10-transformed.

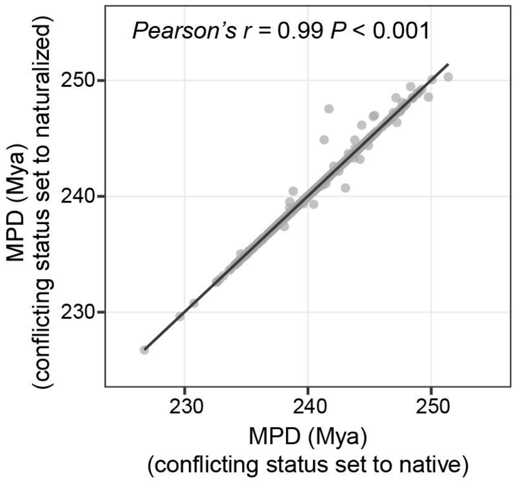

Supplementary Fig. 9. The strong correlation between regional MPD values when species with conflicting statuses are assigned as either native or naturalized in the 487 regions. The correlation was calculated as Pearson's correlation coefficient. The line represents the fitted relationships from a linear regression model.

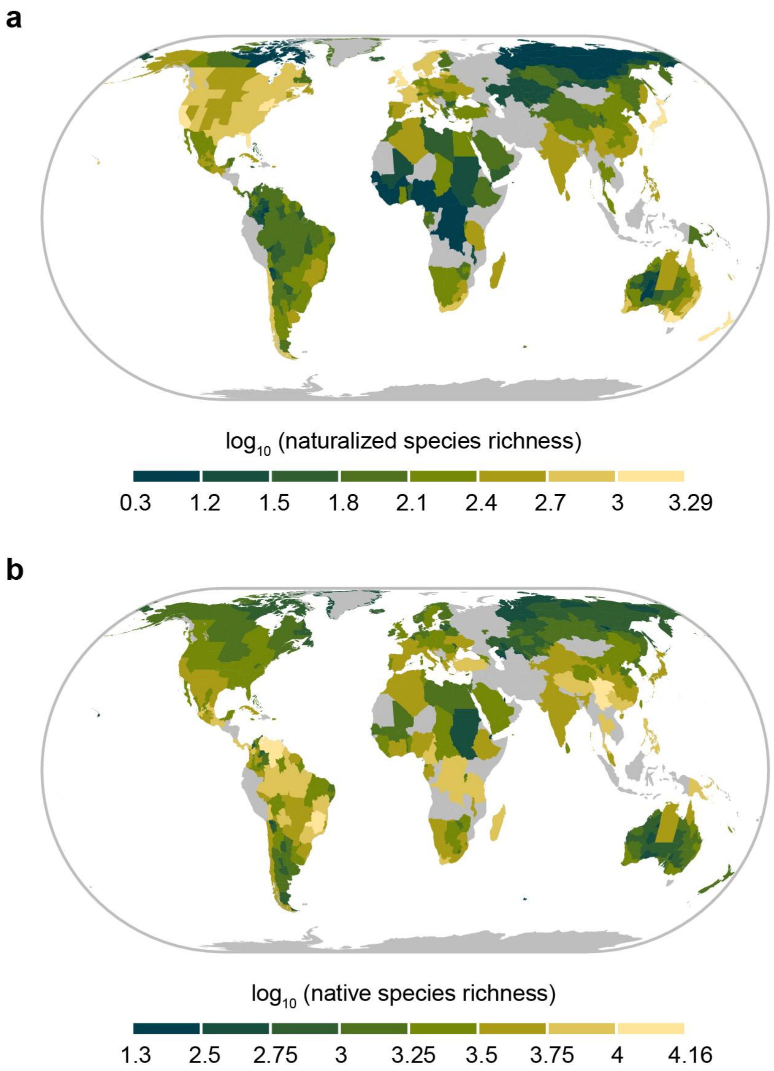

Supplementary Fig. 10. Global map showing the numbers of naturalized species (a) and native species (b) in the 487 regions. Gray areas are regions without data. Note that the numbers of species were log10-transformed.

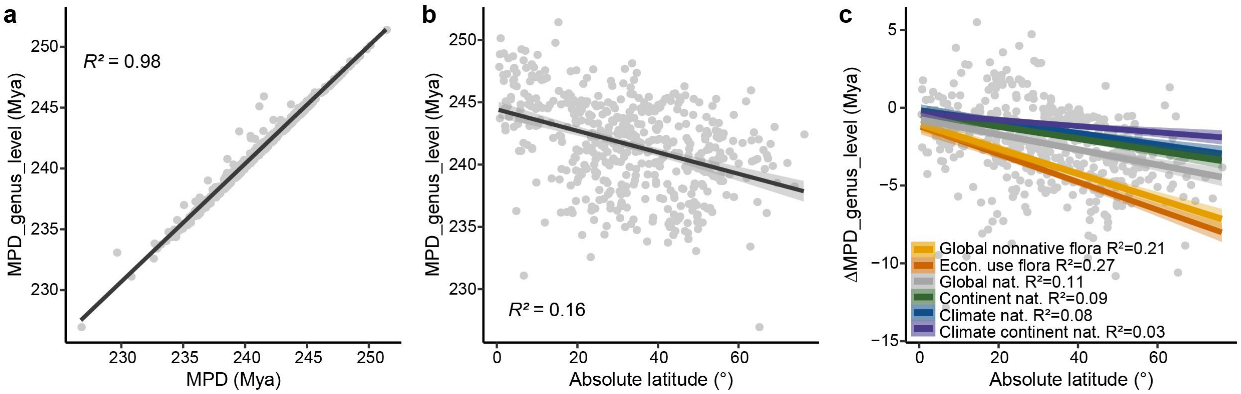

Supplementary Fig. 11. Relationships of MPD and ΔMPD with latitude using a genus-level phylogenic tree in the 487 regions. In the genus-level tree, all species were included as polytomies of their genera. **a** shows the comparison of the MPD values of the regions calculated from the genus-level tree (MPD_genus_level) with the MPD values from the partially resolved species-level tree used in the main text. **b** and **c** show the latitudinal pattern of MPD and ΔMPD values that calculated from the genus-level tree, respectively, In **c**, the plotted dots refer to the null model based on the global naturalized alien species pool, and different colors for the regression lines represent different species pools used to calculate ΔMPD (Supplementary Fig. 2). Lines and shaded areas represent predictions and their 95% confidence intervals, respectively. All relationships are statistically significant ($P < 0.05$), and all R^2 values have been adjusted.

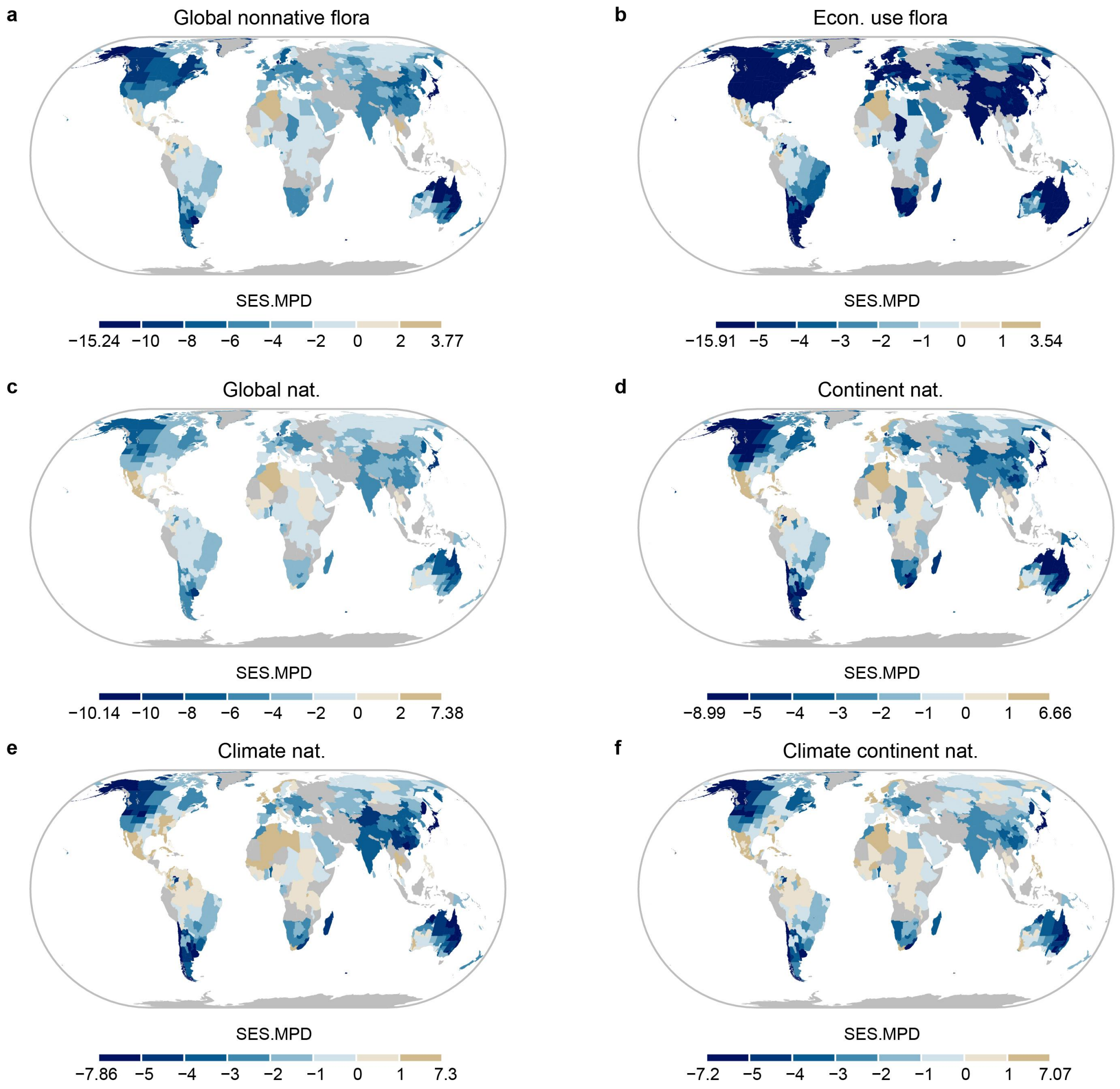

Supplementary Fig. 12. Global maps showing the standardized effect sizes of MPD (SES.MPD) based on six types of null models for the 487 regions. SES.MPDs were calculated using six different species pools (**a**, global nonnative flora; **b**, econ. use flora; **c**, global nat.; **d**, continent nat.; **e**, climate nat.; **f**, climate continent nat.) as shown in Supplementary Fig. 2. Gray areas are regions without data.

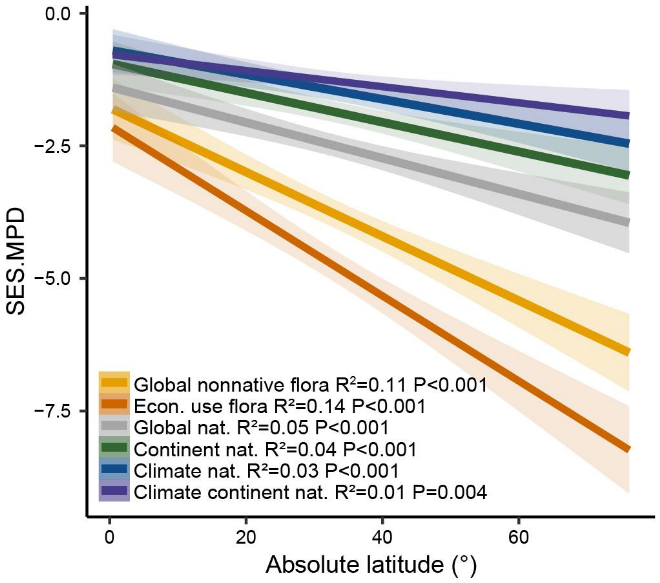

Supplementary Fig. 13. Latitudinal gradient of the SES.MPD for the 487 regions. Different colors represent different species pools used to calculate SES.MPD (Supplementary Fig. 2). Lines and shaded areas represent predictions and their 95% confidence intervals, obtained from the linear models that relate SES.MPD to absolute latitude. All R^2 values have been adjusted.

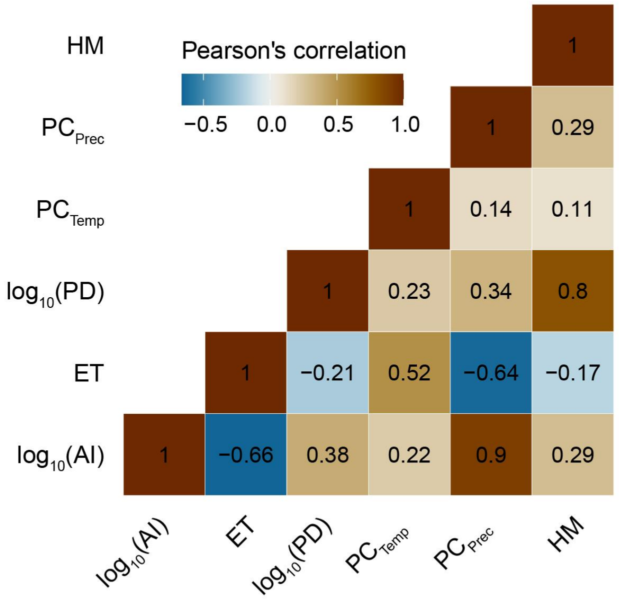

Supplementary Fig. 14. Heatmap of Pearson's correlation analysis between different variables for the 487 regions. Heatmap color keys indicate Pearson's correlation coefficients. The global raster data of the human population density (PD), the global aridity index (AI), and potential evapotranspiration (ET) are available at a resolution of 1-km^2. The average values of these variables for each region were calculated as the mean value across all grid cells in that region. Note that the human population density (PD) and the global aridity index (AI) were log10-transformed.
