# Supplementary material for: A latitudinal gradient in Darwin’s naturalization conundrum at the global scale for flowering plants
Source: Nat Commun. 2023 Oct 12;14:6244. doi: 10.1038/s41467-023-41607-w (PMC10570376; doi:10.1038/s41467-023-41607-w)
Supplement: Supplementary file 3 — Reporting Summary [file 41467_2023_41607_MOESM3_ESM.pdf]

nature portfolio

Corresponding author(s): Shao-peng Li

Last updated by author(s): Aug 28, 2023

Reporting Summary

Nature Portfolio wishes to improve the reproducibility of the work that we publish. This form provides structure for consistency and transparency in reporting. For further information on Nature Portfolio policies, see our Editorial Policies and the Editorial Policy Checklist.

Statistics

For all statistical analyses, confirm that the following items are present in the figure legend, table legend, main text, or Methods section.

n/a	Confirmed	
☐	☒	The exact sample size (*n*) for each experimental group/condition, given as a discrete number and unit of measurement
☒	☐	A statement on whether measurements were taken from distinct samples or whether the same sample was measured repeatedly
☐	☒	The statistical test(s) used AND whether they are one- or two-sided<br>*Only common tests should be described solely by name; describe more complex techniques in the Methods section.*
☒	☐	A description of all covariates tested
☒	☐	A description of any assumptions or corrections, such as tests of normality and adjustment for multiple comparisons
☐	☒	A full description of the statistical parameters including central tendency (e.g. means) or other basic estimates (e.g. regression coefficient) AND variation (e.g. standard deviation) or associated estimates of uncertainty (e.g. confidence intervals)
☐	☒	For null hypothesis testing, the test statistic (e.g. *F*, *t*, *r*) with confidence intervals, effect sizes, degrees of freedom and *P* value noted<br>*Give P values as exact values whenever suitable.*
☒	☐	For Bayesian analysis, information on the choice of priors and Markov chain Monte Carlo settings
☒	☐	For hierarchical and complex designs, identification of the appropriate level for tests and full reporting of outcomes
☐	☒	Estimates of effect sizes (e.g. Cohen's *d*, Pearson's *r*), indicating how they were calculated

Our web collection on statistics for biologists contains articles on many of the points above.

Software and code

Policy information about availability of computer code

Data collection	The GloNAF database together with the shapefile that was used to produce the maps have been published in a data paper. The GIFT database is accessible via the GIFT R-package version 1.1.0 (https://CRAN.R-project.org/package=GIFT).
Data analysis	All the data analysis were performed using R version 4.0.3. We used the R package "Taxonstand" (version 2.4) to standardize the taxonomic names to The Plant List. We calculated the phylogenetic metrics using the R package "picante" (version 1.8.2). We used the R package "normalizer" (0.1.0) to make the bioclimatic variable best approximate a normal distribution. We used the R package "dismo" (1.3.5) to contruct the maximum entropy modelling. We used the R package "visreg" (version 2.7.0) to visualize the effect of the interaction for the model. We performed linear mixed-effects models and a binomial generalized linear mixed model by using the R package "lme4" (version 1.1.28). We calculated the standardized effect sizes of variables using the R package "effectsize" (version 0.6.0.1). The code for the analyses and figures are available at figshare (https://doi.org/10.6084/m9.figshare.20055611.v5).

For manuscripts utilizing custom algorithms or software that are central to the research but not yet described in published literature, software must be made available to editors and reviewers. We strongly encourage code deposition in a community repository (e.g. GitHub). See the Nature Portfolio guidelines for submitting code & software for further information.

Data

Policy information about availability of data

All manuscripts must include a data availability statement. This statement should provide the following information, where applicable:

- Accession codes, unique identifiers, or web links for publicly available datasets
- A description of any restrictions on data availability
- For clinical datasets or third party data, please ensure that the statement adheres to our policy

The data generated in this study have been deposited in the figshare (https://doi.org/10.6084/m9.figshare.20055611.v5). The GloNAF database together with the shapefile that was used to produce the maps have been published in a data paper (van Kleunen et al. 2019, DOI:10.1002/ecy,2542). The GIFT database is accessible via the GIFT R-package (https://CRAN.R-project.org/package=GIFT).

Human research participants

Policy information about studies involving human research participants and Sex and Gender in Research.

Reporting on sex and gender	Not used in this study
Population characteristics	Not used in this study
Recruitment	Not used in this study
Ethics oversight	Not used in this study

Note that full information on the approval of the study protocol must also be provided in the manuscript.

Field-specific reporting

Please select the one below that is the best fit for your research. If you are not sure, read the appropriate sections before making your selection.

☐ Life sciences ☐ Behavioural & social sciences ☒ Ecological, evolutionary & environmental sciences

For a reference copy of the document with all sections, see nature.com/documents/nr-reporting-summary-flat.pdf

Ecological, evolutionary & environmental sciences study design

All studies must disclose on these points even when the disclosure is negative.

Study description	By integrating databases of the global distribution of native and naturalized species, we studied the global pattern and mechanisms of phylogenetic distance between native and naturalized species. Our dataset included the native distributions for 219,520 angiosperms, naturalized alien distributions for 9,531 angiosperms across 487 regions, which represent ~70% of the global angiosperm flora. With these data, we tested the latitudinal variation in naturalized alien-to-native phylogenetic distances, and analysed the impact of climatic and anthropogenic variables on the global pattern.
Research sample	We used existing databases. In our study, the regional lists of naturalized species were extracted from the Global Naturalized Alien Flora (GloNAF) database; the regional lists of native species were extracted from the Global Inventory of Floras and Traits (GIFT) database and other datasets, including the R package GIFT (Weigelt, Patrick, and Pierre Denelle. "GIFT: Access to the Global Inventory of Floras and Traits (GIFT)," March 15, 2023); the Euro+Med PlantBase (http://ww2.bgbm.org/EuroPlusMed/ [accessed 2019/12/30].); the flora checklist of New Guinea (Cámara-Leret et al. 2020, Nature, DOI: 10.1038/s41586-020-2549-5); World Checklist of Vascular Plants (WCVP, https://doi.org/10.34885/nswv-8994). The phylogenetic tree including all angiosperms in our dataset, was developed based on the mega phylogeny of Smith and Brown (2018, American Journal of Botany, DOI: 10.1002/ajb2.1019).
Sampling strategy	Given the scale of our study being global study, we used all the data available from the global datasets.
Data collection	The GloNAF and GIFT database were developed by the authors. Other datasets of global flora and explanatory variables in our study were downloaded by S.Y.F. The phylogeny was developed by Q.Y. based on the phylogenetic tree by Smith and Brown (2018, American Journal of Botany, DOI: 10.1002/ajb2.1019).
Timing and spatial scale	We downloaded the GloNAF and GIFT database on September 2020. We downloaded the regional native species lists from R package GIFT on 17 July 2023, from WCVP on 27 October 2022, and from the Euro+Med PlantBase on 30 December 2019. We downloaded the WorldClim database on 29 March 2022, downloaded the global human modification index on 19 October 2021, and download data of aridity index, potential evapotranspiration, and population density on 28 December 2022. We downloaded the phylogeny on 18 January 2019. The spatial scale of all datasets used in our study is global.
Data exclusions	To align the flora lists from the different databases, we standardized the taxonomic names of all species according to The Plant List,

using the R package Taxonstand v.2.4. To reduce influences of spatial scale on latitudinal pattern, and to make results from different regions more comparable, we restricted our analysis to regions larger than 5,000 km2.

Reproducibility	Our main results are reproducible given the data and R code we used are full accessible through Figshare (https://doi.org/10.6084/m9.figshare.20055611.v5)
Randomization	Randomization does not apply as we analyzed existing databases without experiments.
Blinding	Blinding does not apply as we analyzed existing databases without experiments.

Did the study involve field work? ☐ Yes ☒ No

Reporting for specific materials, systems and methods

We require information from authors about some types of materials, experimental systems and methods used in many studies. Here, indicate whether each material, system or method listed is relevant to your study. If you are not sure if a list item applies to your research, read the appropriate section before selecting a response.

Materials & experimental systems

n/a	Involved in the study	
☒	☐	Antibodies
☒	☐	Eukaryotic cell lines
☒	☐	Palaeontology and archaeology
☒	☐	Animals and other organisms
☒	☐	Clinical data
☒	☐	Dual use research of concern

Methods

n/a	Involved in the study	
☒	☐	ChIP-seq
☒	☐	Flow cytometry
☒	☐	MRI-based neuroimaging
